# Quality of life and its predictors among patients with chronic kidney disease: A hospital-based cross sectional study

**DOI:** 10.1371/journal.pone.0212184

**Published:** 2019-02-27

**Authors:** Belayneh Kefale, Minyahil Alebachew, Yewondwossen Tadesse, Ephrem Engidawork

**Affiliations:** 1 Department of Pharmacy, College of Medicine and Health Science, Ambo University, Ambo, Ethiopia; 2 Department of Pharmacology and Clinical Pharmacy, School of Pharmacy, College of Health Sciences, Addis Ababa University, Addis Ababa, Ethiopia; 3 Department of Internal Medicine, School of Medicine, College of Health Sciences, Addis Ababa University, Addis Ababa, Ethiopia; University of Mississippi Medical Center, UNITED STATES

## Abstract

**Introduction:**

Quality of life (QoL) is increasingly being considered as an important measure of how disease affects patients’ lives, especially for long-term diseases like chronic kidney disease (CKD). Even though there is no statistically significant association between stages of CKD and QoL, it is decreased in patients with early stages of the disease. Hence, this study aimed to assess QoL and its predictors among patients with CKD at Tikur Anbessa Specialized Hospital (TASH).

**Methods:**

A cross sectional study was conducted at the nephrology clinic of TASH. A total of 256 patients were recruited through systematic random sampling. Data were collected using the Medical Outcomes Study Short Form 36-Items (SF-36). The data were entered into Epi Info 7.2.2.2 and analyzed using SPSS version 20.0 statistical software. Descriptive statistics like frequency, percent, mean and standard deviation were used to summarize patients’ baseline characteristics. Student’s unpaired t-test and ANOVA were conducted to compare two groups and more than two groups in the analysis of QoL, respectively. Multivariable linear regression was employed to investigate the potential predictors of QoL.

**Results:**

Quality of life was decreased in all stages of CKD. A reduction in physical functioning (p = 0.03), bodily pain (p = 0.004), vitality (p = 0.019) and social functioning (p = 0.002) was observed progressively across stages of CKD. High income status and greater than 11g/dl hemoglobin level were found to be predictors of all high score SF-36 domains. High family income (β 15.33; 95%CI: 11.33–19.33, p<0.001), higher educational status (β 7.9; 95%CI: 4.10–11.66, p<0.001), and hemoglobin ≥11g/dl (β 8.36; 95%CI: 6.31–10.41, p<0.001) were predictors of better QoL in the physical component summary, whereas absence of CKD complications (β 2.75; 95%CI: 0.56–4.94, p = 0.014), high family income (β 10.10; 95%CI: 5.10–15.10, p<0.001) and hemoglobin ≥11g/dl (β 4.54, 95%CI: 2.01–7.08, p = 0.001) were predictors of better QoL in the mental component summary.

**Conclusion:**

In this setting, QoL decreased in CKD patients in the early stages of the disease. Study participants with low income and hemoglobin level were considered to have worse quality of life in both physical and mental component summaries.

## Introduction

Chronic kidney disease (CKD) is defined as abnormal kidney structure or function persisting greater than 3 months [1]. This can be determined either by evidence of kidney damage (typically detected by presence of persistent albuminuria) or by decreased glomerular filtration rate (GFR)[2, 3]. CKD is a global public health problem due to the rapid rise of common risk factors such as diabetes and hypertension. Consequently, it will result significance menace to the developing nations with limited resources [2]. Its burden on health care system is becoming immense with increasing prevalence, high costs, and poor outcomes [4]. CKD is associated with increased risks of cardiovascular morbidity, premature mortality, and has severe impact on quality of life (QoL) [4]. Mortality from cardiovascular disease (CVD) is estimated to be at least 8 to 10 fold higher in CKD patients as compared to non-CKD patients [4]. The costs involved in the management CKD co-morbidities are very high, imposing great difficulties on health care systems, predominantly in countries with limited resources [5].

Studies demonstrated that CKD patients living with significant constraints and restrictions might have an impaired normal psychosocial development [6, 7]. QoL is an independent risk factor for mortality in CKD patients particularly in end stage renal disease (ESRD) [8, 9]. Moreover, several factors such as disease related manifestations, side effects of treatments, and patient’s quality of interaction with family members can influence QoL [10, 11]. Low QoL have been the major problems in CKD patients and their occurrence can adversely impact the course of the disease [12, 13]. Patients with CKD have a reduced QoL and an increased frequency and severity of both symptoms and psychological distress, with the magnitude of these changes negatively correlated with GFR [6, 10]. Association of poorer QoL with preventable factors demonstrated that attention should be given to psychosocial and medical interventions to improve QoL in CKD patients [11]. The available data on QoL of patients on conservative treatment and the relationship between the QoL and GFR is limited [12, 13]. Even though patients with advanced renal insufficiency have a reduced QoL, some studies showed that QoL is decreased in the early stages of disease [14].

The incidence of CKD in Ethiopia is rising because of increased risk factors such as hypertension and diabetes mellitus [15]. An extensive study of factors affecting QoL would render valuable perspicacity for the healthcare providers in renal clinic to improve QoL. However, evidence-based research that evaluates QoL and associated factors among patients with CKD in developing countries is scanty [9]. Thus, there should be a continuing need to routinely assess factors affecting QoL among patients with CKD in clinical practice [11, 16]. This is especially important in resource-limited countries like Ethiopia, as the preponderance of economic instability, low literacy level, and restricted access to healthcare facilities, inadequate follow up and co-morbidities might have led to worse QoL. Therefore, this study aimed to evaluate the quality of life (QoL) of CKD patients at Tikur Anbessa Specialized Hospital and to determine the related factors.

## Methods

### Study design and settings

A cross-sectional study was conducted from May to September 2017 at the nephrology clinic of Tikur Anbessa Specialized Hospital (TASH), the largest general public hospital, where tertiary care is being provided in Ethiopia. The Nephrology clinic provides treatment to different types of renal disease and its complications. The renal days are mainly Monday, Tuesday and Thursday; on an average 6, 12 and 12 CKD patients were selected, respectively. Single population proportion formula was used to calculate the sample size [17]

n=Zα22p(1−p)d2

Where;

*n* = is desired sample size for population >10,000;

*Z* = is standard normal distribution usually set as 1.96 (which corresponds to 95% confidence level);

*P* = means that we use positive prevalence estimated, to maximize sample size. Negative prevalence = 1 − 0.5 = 0.5,

d = degree of accuracy desired (marginal error is 0.05); then the sample size is

$$n=\frac{1.96^{2}0.5(1-0.5)}{(0.05{)}^{2}}=384.16=∼384$$

The expected number of source population in the study period (N), based on the average number of patients coming to the clinic three days in a week with a total of 20 weeks was 600 (20*6+20*12+20*12).

$$Corrected\;sample\;size=\frac{n\times N}{n+N}$$

Where N = source population and n = estimated sample size for N≥10,000 population.

$$Corrected\;sample\;size=\frac{384\times 600}{384+600}=233$$

Therefore, the corrected sample size with a 10% contingency provided a final sample size of 256 CKD patients.

### Inclusion criteria

CKD patients aged 18 years and above and who understood Amharic language were targeted since Amharic is the predominant and national language in the region. However, patients with cognitive, speech or hearing impairment were excluded from the study and only those who signed the informed consent were included in the study.

### Data collection techniques

Three nurses were recruited as data collectors. Training was given to them regarding appropriate use of the data collection instruments focusing on uniform interpretation of questions, strict use of study criterion, explanation of study objectives and getting consents from study participants.

Data were collected using structured questionnaire, which contained socio-demographic characteristics, clinical parameters and The Medical Outcomes Study Short Form 36-Item Health Survey (SF-36) (S1 Annex). SF-36 is a generic instrument translated and validated scale in Amharic [18], was utilized to collect information necessary to assess QoL. The 36-item short form questionnaire is a multi-item scale, not specific to any disease or treatment group. The SF-36 is covered by a conceptual model of QoL [19] and includes 36 items that yield an 8-dimension profile on a 100-point scale; a higher score indicates a better perceived health state. The eight dimensions are: Physical function (PF), Role limitations caused by physical problems (RP), Pain (BP), General health (GH), Vitality/energy (VT), Social function (SF), mental health/emotional well-being (MH) and Role limitations caused by emotional problems/mental health (RE). In addition it is used to estimate change in health status over the past year. The data collection instrument was assessed by an expert physician in the field of nephrology for clarity and comprehensiveness of its contents. Pre-testing was done on 5% of the study participants before the start of the actual study. After pre-testing all the necessary modifications and adjustments were done before implementing in the main study.

### Data analysis

Data were sorted, cleaned, coded and entered into Epi Info 7.2.2.2 and analyzed using SPSS version 20.0 statistical software. Descriptive statistics such as frequency, percent, mean and standard deviation were used to summarize patients’ baseline socio-demographic data and clinical parameters. Student’s unpaired t-test and one-way ANOVA were conducted to compare two groups and three or more groups in the analysis of QoL, respectively. When t-test & ANOVA result was statistically significant, multivariable linear regression was employed to identify the potential predictors of the physical and mental component summary. P-value < 0.05 considered as statistically significant.

## Results

### Socio-demographic characteristics

Males comprised 58% of the sex category. Majority of the participants were in the age group of less than 61 years, which accounted for 54.3%. Mean age of the study population was 52.5 (SD = 16.8) years (range 18 to 90 years). Married participants accounted for 69.9% and being retired (25.4%) and government employee (23.4%) accounted for the highest percentage of occupation. Education-wise, 34.4% and 27.7% attended primary and higher education, respectively. Majority of the participants were non-health professionals (97.3%). A significant proportion of the study participants (29.7%) had average level of monthly family income [20] (Table 1).

**Table 1:** Socio-demographic characteristic of chronic kidney disease patients attending the renal clinic of Tikur Anbessa Specialized Hospital.

Variables	Stage of CKD				
	1 & 2 (n = 50)	3 (n = 88)	4 (n = 55)	5 (n = 63)	Total (n = 256)
**Sex**					
Male	25 (50)	60 (68.2)	31 (56.4)	33 (52.4)	149 (58)
Female	25 (50)	28 (31.2)	24 (43.6)	30 (47.6)	107 (42)
**Age (years)**					
≤60	38 (76)	41 (46.6)	28(50.9)	32(50.8)	139 (54.3)
>60	12(24)	47 (53.4)	27(49.1)	31(49.2)	117 (45.7)
**Marital status**					
Single✞	14(28)	23(26.1)	20(36.4)	20(31.7)	77 (30.1)
Married	36(72)	65(73.9)	35(63.6)	43(68.3)	179 (69.9)
**Occupation**					
Farmer	6(12)	8(9.1)	4(7.3)	6(9.5)	24 (9.4)
Gov’t employee	18(36)	19(25.6)	11(20)	12(19.1)	60 (23.4)
Merchant/trade	7(14)	5(5.7)	5(9.1)	6(9.5)	23 (9)
Daily laborer	2(4)	6(6.8)	4(7.3)	7(11.1)	19 (7.4)
Housewife	7(14)	11(12.5)	8(14.5)	11(17.5)	37 (14.5)
Retired	6(12)	27(30.7)	18(32.7)	14(22.2)	65 (25.4)
Others*	4(8)	12(13.6)	5(9.1)	7(11.1)	28 (10.9)
**Profession**					
Health professional	3(6)	1(1.1)	2(3.6)	1(1.6)	7 (2.7)
Non-health professional	47(94)	87(98.9)	53(96.4)	62(98.4)	249 (97.3)
**Educational status**					
Cannot read and write	5(10)	11(12.5)	7(12.7)	7(11.1)	30 (11.7)
Primary	13(26)	31(35.23)	20(36.4)	24(38.1)	88 (34.4)
Secondary	10(20)	23(26.1)	19(34.5)	15(23.8)	67 (26.2)
Higher Education	22(44)	23(26.1)	9(16.4)	17(27)	71 (27.7)
Monthly family income (ETB)**					
Very low (≤860)	4(8)	10(11.4)	11(20)	15(23.8)	40 (15.6)
Low (861–1500)	13(26)	21(23.9)	17(30.9)	21(33.3)	72 (28.1)
Average (1501–3000)	10(20)	33(37.5)	18(32.7)	15(23.8)	76 (29.7)
Above average (3001– 5000)	17(34)	20(22.7)	6(10.9)	8(12.7)	51 (19.9)
High (≥5001)	6(12)	4(4.5)	3(5.5)	4(6.4)	17 (6.7)

✞Single, divorced and widowed

*students, driver, garage (mechanic), guard, teacher working in private school

** Based on the Ethiopian Civil Service monthly salary scale for civil servants

### Disease related characteristics

Overall, patients had been diagnosed with CKD for an average of 4.7 (SD = 3.5) years, ranging from under five years (158, 61.7%) through 5–10 years (75, 29.3%) to above ten years (23, 9%). In stage 5 CKD patients, having more than three co-morbidities (47.5%) and complications (66.7%) were the most prevalent clinical conditions. Fasting blood sugar, serum creatinine and blood urea nitrogen increased, while hemoglobin decreased across the stages (Table 2).

**Table 2:** Clinical and laboratory parameters according to the stage of chronic kidney disease patients attending the renal clinic of Tikur Anbessa Specialized Hospital.

Clinical/laboratory parameters	Stage of CKD				
	1 & 2 (n = 50)	3 (n = 88)	4 (n = 55)	5 (n = 63)	Total (n = 256)
Number of co-morbidities					
≤ 2	43 (20.8)	77 (37.2)	46 (22.2)	41 (19.8)	207 (100)
3 or more	4 (10)	8 (20)	9 (22.5)	19 (47.5)	40 (100)
Number of complications					
≤ 2	9 (10.2)	29 (33)	23 (26.1)	27 (30.7)	88 (100)
3 or more	0	0	1 (33.3)	2 (66.7)	3 (100)
FBS (mg/dl)	125 ± 46	140 ± 46	149 ± 69	155 ± 57	141 ± 56
Scr(mg/dl)	1.6 ± 0.8	2.0 ± 0.8	3.5 ± 1.6	7.6 ± 3.1	3.6 ± 3
BUN(mg/dl)	41 ± 20	56 ± 34	93 ± 46	136 ± 66	80 ± 57
Hgb(gm/dl)	16.0 ± 18.9	13.6 ± 14.1	10.5 ± 2.6	10.3 ± 2.8	12.6 ± 12.0
MAP(mmHg)	104.9 ± 12.2	101.7 ± 9.6	104.3 ± 14.3	103.6 ± 14.3	103.4 ± 12.4
GFR(ml/min/1.73m^2^)	74.7 ± 15.4	43.3 ± 8.4	23 ± 4.8	10.4 ± 2.9	37 ± 24.2

FBS = fast blood sugar, Scr = serum creatinine, BUN = blood urea nitrogen, Hgb = hemoglobin, MAP = mean arterial pressure and GFR = glomerular filtration rate

### Quality of life SF-36 domains and summary scores

QoL, as evaluated by the means of SF-36 domains, scores in all dimensions were impaired progressively and significantly (p<0.05) across CKD stages and the lowest scores were found in stage 5 CKD patients except emotional role functioning in stage 4 (p = 0.005). As shown in Table 3, the dimensions showing highest and least scores were emotional role functioning (78±34.7) and bodily pain (59.3±22.6) in stages 1 and 2, social functioning (68±24.6) and physical role functioning (48.6±40.4) in stage 3, mental health (55.6±18.9) and physical role functioning (38.2±40.2) in stage 4 and emotional role functioning (56.1±45.6) physical role functioning (26.6±40.4) in stage 5, respectively (Table 3).

**Table 3:** Quality of life domains and mean summary scores of patients in different stages of chronic kidney disease patients attending the renal clinic of Tikur Anbessa Specialized Hospital.

Scales	No of items	Cronbach’s α	Stages of CKD				
			1 & 2 (n = 50)	3 (n = 88)	4 (n = 55)	5 (n = 63)	Total (n = 256)
**PF**	10	0.831	65.1±17.5	60.8±22.6	54.5±25.9	51.3±28.1	57.9±24.4
**RP**	4	0.834	66±37.7	48.6±40.4	38.2±40.2	26.5±40.4	44.3±41.9
**BP**	2	0.837	59.3±22.6	58.8±25	40.9±23.8	35.3±24.1	49.3±26.2
**GH**	5	0.846	61.4±16.2	55±17	46.4±18.9	45.4±18.5	52±18.6
**VT**	4	0.841	66.9±15.4	65.1±16.2	54.2±19.2	52.5±14.5	60±17.5
**SF**	2	0.842	68.6±19.6	68±24.6	54.7±26.5	45.5±22.5	59.7±25.4
**RE**	3	0.870	78±34.7	61.4±45.5	49.7±42.5	56.1±45.6	60.8±43.8
**MH**	5	0.840	67.4±14.4	67.7±15.8	55.6±18.9	54.3±16.8	61.8±17.6
**PCS**	-	-	42.5±7.5	40±8.8	36.3±9	33.4±10.1	38.1±9.5
**MCS**	-	-	49.7±6.9	48.4±8.9	42.6±9.3	42.8±8.9	46±9.1

PF = Physical functioning, RP = Physical role functioning, BP = Bodily pain, GH = General health, VT = Vitality, SF = Social functioning, RE = emotional role functioning, MH = Mental health, PCS = Physical summary scores, MCS = Mental summary scores.

### Factors associated with quality of life

The results of the comparative statistical analysis of the QoL domains of CKD patients according to the categorical socio-demographic variables are shown in Table 4. Among the domains that constitute the SF-36 physical and mental component summaries, higher scores in all SF-36 domains were observed in patients ≤ 60 years (p<0.05) and higher education (p<0.05) groups. High family income (p<0.001) groups had higher score in all SF-36 domains except physical functioning, emotional role functioning, and mental health. The present study showed that male patients had escalated QoL in terms of general health (p = 0.034), vitality (p = 0.038), social functioning (p = 0.011) and mental health (p = 0.018). In addition, occupation had statistical significant difference with physical functioning (p = 0.001), physical role functioning (p<0.001), bodily pain (p = 0.007) and vitality (p = 0.026) (Table 4).

**Table 4:** Comparative statistical analysis of mean scores of SF-36 domains among patients with chronic kidney disease, according to the categorical socio-demographic variables.

	PF	RP	BP	GH	VT	SF	RE	MH
**Sex**								
Female	54.9±23	41.1±42.4	46.6 ± 24	49.1±18.7	57.3 ± 16	55 ±24.5	59.5 ± 44.2	58.7±16.9
Male	60.1±5.2	46.6 ± 41.5	51.2±7.6	54.1±18.3	61.9±8.3	63.2±5.6	61.8 ± 43.6	63.9±7.8
p-value	0.093	0.299	0.158	0.034	0.038	0.011	0.683	0.018
**Age**								
≤60	64.6±21.4	56.1 ± 41.4	53.6±25.8	55.2±17.6	63.3±15.9	63.6 ±3.9	67.6 ± 42	65 ±14.7
>60	50 ± 25.4	30.3 ± 38	44 ± 25.8	48.3±19.1	56 ± 18.4	55.2± 26.5	52.7 ± 44.5	57.8 ±20
p-value	<0.001	<0.001	0.004	0.003	0.001	0.008	0.006	0.001
**Occupation**								
Farmer	57.7±24.8	39.6 ± 40.3	44.2 ±3.5	53.1±9.5	60.2 ±18	54.8±17.6	62.5±45.4	59.8±16.2
Gov’t employee	65.3±17.2	63.3 ±37.5	56.8±23.4	55.1± 19	65.1±15.4	62 ±22.2	71.2±39.5	66.5±12.9
Merchant	62.6±25.5	52.2 ±43.9	53 ±27.7	56.3±16.9	64.3±18.1	60.5±27.8	59.4±42.6	65.6±16.7
Daily laborer	70.8±22.2	51.3 ± 46	58.5±27.5	60.4±14.4	63.4±16.2	69.9±24.7	68.4±42.3	65.7±18.7
Housewife	53.6±2.4	23.1 ± 34	39.1 ±2.2	49.9±16.4	54.3 ± 12	54.6±23.4	63±45	58.3±12.7
Retired	48.5±27.7	32.3 ± 40	44 .2± 28	47.2±19.7	56.3±19.3	58.6±29.6	48.7±45.3	57.9±22.1
Others	57.1±24.6	52.7 ± 42.7	53.5±26.9	49.6±18.7	59.1± 10.1	60.9±27.5	58.4±45	60.9±19.4
p-value	0.001	<0.001	0.007	0.057	0.026	0.412	0.163	0.076
**Education status**								
Cannot read & write	48.2±24.4	18.3±30.7	41.3±27	46.4±18.1	53.7±19.1	43.5±21	48.9±46.1	52.7±16.6
Primary	57.2±25.9	44.3+42.7	46.5±26.1	51.8±17.6	59.5±18.3	58.1±24.8	61±46.4	61.2±18.8
Secondary	55.4±25.7	39.9±42.9	48.3±25	48±18.9	57.2±16.7	60.9±27.8	52.7±43.5	59.9±18.2
Higher education	65.4±18.9	59.5±38.1	57±25.7	58.5±18.1	65.9±14.7	67.6±22.2	73.3±36.8	67.9±13.7
p-value	0.006	<0.001	0.017	0.002	0.003	<0.001	0.015	<0.001
**Family income status**								
Very low	40.8±25.8	10.6±24.6	26.9±19.4	40.6±16.4	41.1±16.8	39.6±24.2	42.5±47.1	46.1±15.8
Low	46.7±22.2	22.9±33	43.3±25.2	46.4±17.2	57.8±17.1	55±26.1	42.1±42	58.8±19.6
Average	68.2±21.8	58.2±42.5	55.8±24.4	55.6±18.4	64.6±12.5	67.1±22.5	70.6±43.9	65.7±14.9
Above average	69.6±15.5	71.1±33.3	60.6±22.7	60.2±16.1	67.8±14.7	66.8±21.4	83±29.4	70.4±12.7
High	69.4±13.3	72.1±27.8	64.2±22.6	62.4±16.8	69.4±12.4	72.9±17.7	72.7±31.7	67.5±9
p-value	<0.001	<0.001	<0.001	<0.001	<0.001	<0.001	<0.001	<0.001

PF = Physical functioning, RP = Physical role functioning, BP = Bodily pain, GH = General health, VT = Vitality, SF = Social functioning, RE = Emotional role functioning, MH = Mental health

Comparative statistical analysis of the SF-36 domains of CKD patients according to the clinical & laboratory parameters are shown in Table 5. Among the domains that constitute the SF-36 physical and mental component summaries, lower scores in SF-36 domains were associated with higher CKD stages (p<0.05) except emotional role functioning, on five or more medications (p<0.001), having three or more co-morbidities (p≤0.001), presence of CKD complications (p<0.001), having hemoglobin ≤11g/dl level (p<0.001) and being non-adherent to their medications (p<0.05). General health was low in patients having greater than 110 mmHg mean arterial pressure (Table 5).

**Table 5:** Comparative statistical analysis of the mean scores of SF-36 domains of patients with chronic kidney disease, according to the categorical clinical/laboratory parameters.

Variables	PF		RP	BP	GH	VT	SF	RE	MH
**CKD stage**									
1 & 2	65.1±17.5		66±37.7	59.3±22.6	61.4±16.2	66.9±15.4	68.6±19.6	78±34.7	67.4±14.4
3	60.8±22.6		48.6±40.4	58.8±25	55±17	65.1±16.2	68±24.6	61.4±45.5	67.7±15.8
4	54.5±25.9		38.2±40.2	40.9±23.8	46.4±18.9	54.2±19.2	54.7±26.8	49.7±42.5	55.6±18.9
5	51.3±28.1		26.6±40.4	35.3±24.1	45.4±18.5	52.5±14.5	45.5±22.5	56±45.6	54.3±16.8
p-value	0.009		<0.001	<0.001	<0.001	<0.001	<0.001	0.007	<0.001
**N****o** **of medications**									
<5	64.1±21.6		51±40.9	52.9±26.1	54.9±17.9	62.2±16.1	64.2±24.6	64.2±42.6	64.7±15.7
≥ 5	44.1±24.7		29.4±40.4	41.1±24.7	45.6±18.5	55.1±19.4	50±24.4	46.4±43.2	55.1±19.8
p-value	<0.001		<0.001	0.001	<0.001	0.003	<0.001	<0.001	<0.001
**Adherence rate**									
Non adhered	49.3±24.6		24.8±36	38.1±23	43.6±16.9	52.8±17.8	49.8±24.7	52.2±45	54.9±18.3
Adhered	63.3±22.7		56.7±40.7	56.4±25.6	57.4±17.6	64.5±15.7	66±23.9	66.3±42.2	66.1±15.8
p-value	<0.001		<0.001	<0.001	<0.001	<0.001	<0.001	0.012	<0.001
**N****o** **of co-morbidity**									
0–2	62.2±22.1		49.5±42.1	51.6±25.9	54.3±17.6	61.5±17.3	62.7±24.6	65.8±43.3	64±16.8
≥ 3	34.6±23.1		16.3±26.9	36.9±24.6	40±19.1	52±16.2	43.6±23.6	34.1±36.6	49.4±17.1
p-value	<0.001		<0.001	0.001	<0.001	0.001	<0.001	<0.001	<0.001
**Complications**									
Present	47.1±25		29.7±38.9	40.1±25.4	46.2±18.4	54±+18	50.2±25.9	46.8±44.7	53.7±18.8
Absent	63.9±22		52.4±41.3	54.4±25.3	55.2±18	63.3±16.3	65±23.6	68.5±41.4	66.2±15.2
p-value	<0.001		<0.001	<0.001	<0.001	<0.001	<0.001	<0.001	<0.001
**Hemoglobin**									
≤11g/dl	40.6±21.7		12.1±24.4	32.5±21.6	41.1±16.9	50±17.2	46.7±25.4	36.1±42.5	51.9±17.9
>11g/dl	70.4±17.6		67.4±36.1	61.3±22.3	59.9±15.6	67.1±13.8	69.1±20.9	78.6±35.3	68.8±13.6
p-value	<0.001		<0.001	<0.001	<0.001	<0.001	<0.001	<0.001	<0.001
**MAP**									
≤110 mmHg	58.2±24.8		45.4±41.4	50.6±26.2	53.1±18.8	60.6±17.6	60.2±25.8	60.5±43.6	62.2±17.7
>110 mmHg	56.6±22.6		39.2±44.3	43.2±25.4	47±16.6	56.9±16.6	57.6±23.8	62.1±45.3	59.5±17.1
p-value	0.690		0.373	0.089	0.046	0.201	0.542	0.828	0.362

Pf = Physical functioning, RP = Physical role functioning, BP = Bodily pain, GH = General health, VT = Vitality, SF = Social functioning, RE = Emotional role functioning, MH = Mental health, MAP = mean arterial pressure

According to the comparative statistical analysis of categorical socio-demographic variables across composite summary, higher scores in the physical and mental component summary were found among patients who were younger (p<0.001, p<0.05), had higher education (p<0.001, p<0.001) and high family income (p<0.001, p<0.001), respectively of CKD patients. Occupation had also statistically significant mean difference with physical component summary (Table 6).

**Table 6:** Comparative statistical analysis of the mean scores of physical and mental composite summaries of patients with chronic kidney disease, according to the categorical socio-demographic variables.

Variables	PCS	p-value	MCS	p-value
**Sex**				
Female	36.9 ± 9.5	0.090	44.7 ± 9	0.051
Male	38.9 ± 9.5		47 ± 9.1	
**Age**				
≤60	40.7 ± 8.9	<0.001	47.3 ± 8.1	0.012
>60	34.9 ± 9.2		44.5 ± 10	
**Occupation**				
Farmer	37.1 ± 9.4	<0.001	45.6 ± 8.1	0.538
Gov’t employee	41.7 ± 7.8		47.8 ± 8	
Merchant	40.5 ± 10.2		46.5 + 9.3	
Daily laborer	42.5 ± 9.5		47.7 ± 8.6	
Housewife	33.7 ± 8.5		45.8 ± 7.2	
Retired	34.9 ± 9.7		44.5 ± 11	
Others	39.3 ± 9		45.1 ± 10	
**Education status**				
Cannot read and write	33.6 ± 9.4	<0.001	41.7 ± 8.3	<0.001
Primary	37.6 ± 9.7		45.8 ± 9.5	
Secondary	37.2 ± 9.4		44.8 ± 9.6	
Higher Education	41.4 ± 8.5*		49.3 ± 7.5*	
**Family income status**				
Very Low	29.7 ± 6.8	<0.001	38.8 ± 9.2	<0.001
Low	33.5 ± 8.3		44.3 ± 9.1^œ^	
Average	41.7 ± 8.7^⌖^		48 ± 8.6^⌖^	
Above Average	43.3 ± 6.7^⌖^		50.3 ± 6.5^⌖^	
High	45.1 ± 7^⌖^		48.9 ± 6.8^⌖^	

PCS = Physical summary scores, MCS = Mental summary scores

*p<0.001 compared with cannot read and write

^œ^p = 0.004 compared with very low family income

^⌖^p<0.001 compared with very low family income

As per the comparative statistical analysis of clinical & laboratory parameters, lower scores in the physical and mental component summary were found among patients who were on advanced CKD stage (p<0.001, p<0.001), five or more medications (p<0.001, p<0.001), three or more co-morbidities (p<0.001, p<0.001) and had hemoglobin ≤11g/dl level (p<0.001, p<0.001), respectively of CKD patients (Table 7).

**Table 7:** Comparative statistical analysis of mean scores of physical and mental composite summaries of patients with chronic kidney disease, according to the categorical clinical & laboratory parameters.

Variables	PCS	p-value	MCS	p-value
**CKD stage**				
1 & 2	42.5 ± 7.5	<0.001	49.7 ± 6.9	<0.001
3	40 ± 8.8		48.4 ± 8.9	
4	36.3 ± 9^✞^		42.6 ± 9.3*	
5	33.4 ± 10.1*		42.8 ± 8.9*	
**Number of medications**				
<5	40 ± 9	<0.001	47.4 ± 8.2	<0.001
≥ 5	33.7 ± 9.3		42.9 ± 10.3	
**Adherence rate**				
Non-adhered	33.2 ± 8.1	<0.001	43.2 ± 9.6	<0.001
Adhered	41.2 ± 9		47.8 ± 8.4	
**Number of co-morbidity**				
0–2	39.4 ± 9.1	<0.001	47.1 ± 9	<0.001
≥ 3	30.7 ± 8.4		40.3 ± 8	
**Complications**				
Present	34.3 ± 9.2	<0.001	42.2 ± 9.3	<0.001
Absent	40.1 ± 9		48.1 ± 8.4	
**Hemoglobin**				
Hgb≤ 11g/dl	30.5 ± 6.5	<0.001	41.1 ± 9.1	<0.001
Hgb>11g/dl	43.5 ± 7.3		49.5 ± 7.4	
**MAP**				
MAP≤110 mmHg	38.5 ± 9.6	0.112	46.1 ± 9.2	0.740
MAP>110 mmHg	36 ± 8.9		45.68 ± 8	

PCS = Physical summary scores, MCS = Mental summary scores, MAP = mean arterial pressure

*p < 0.001 compared with CKD stage 1 & 2

^✞^p = 0.001 compared with CKD stage 1 & 2

After adjustment through multivariable linear regression, higher family income status and greater than 11g/dl hemoglobin level were found to be predictors of all high score SF-36 domains. Being female, presence of complications, advanced stage of CKD, patients with five or more medications and three or more co-morbidities were predictors of low physical functioning. Being adherent to medications and absence of CKD complications were found predictors of better general health and mental health, respectively. Likewise, advanced CKD was a predictor of worse social functioning, vitality and bodily pain (Table 8). No associations were detected between any other socio-demographic or clinical variables and the scores of the SF-36.

**Table 8:** Adjusted analysis for predictors of SF-36 domains among chronic kidney disease patients.

SF-36 domains		Coefficients (95% CI)	p-value
**Physical functioning**			
Male		5.0[0.28–9.72]	0.038
Absence of complications		5.05 [0.16–9.94]	0.043
≥ 3 co morbidity		-11.44 [-18.23-(-4.6)]	0.001
CKD stages	1 & 2	1.00	
	3	-4.3 [-11.6-(3.0]	0.247
	4	-10.6 [-19.3-(-2.0]	0.016
	5	-13.8 [-22.9-(-4.8]	0.003
Family income status	Very low	1.00	
	Low	4.9 [-4.25–14.14]	0.289
	Average	27.4 [18.4–36.4]	<0.001
	Above average	28.9 [20.2–37.5]	<0.001
	High	28.7 [15.3–42.0]	<0.001
≥ 5 medications		-7.9 [-13.27-(-2.53)]	0.004
Hgb>11g/dl		21.5 [15.8–27.14]	<0.001
**Role Limitation physical**			
Family income status	Very low	1.00	
	Low	12.3[0.5–24.1]	0.042
	Average	47.6[33.1–62.1]	<0.001
	Above average	60.5[47.9–73.0]	<0.001
	High	61.4[46.6–76.3]	<0.001
Hgb>11g/dl		37.42 [27.79–47.05]	<0.001
**Bodily pain**			
CKD stages	1 & 2	1.00	
	3	-0.4 [-8.9–8.0]	0.098
	4	-18.3 [-27.3-(-9.3)]	<0.001
	5	-23.9 [-32.7-(-15.1)]	<0.001
Family income status	Very low	1.00	
	Low	16.4 [7.3–25.5]	0.001
	Average	28.9 [20.1–37.8]	<0.001
	Above average	33.8 [24.8–42.7]	<0.001
	High	37.4 [25.5–49.2]	<0.001
Hgb>11g/dl		16.5 [9.72–23.3]	<0.001
**General health**			
Family income status	Very low	1.00	
	Low	5.8 [-0.8–12.4]	0.087
	Average	15.0 [8.1–21.9]	<0.001
	Above average	19.6 [12.8–26.4]	<0.001
	High	21.8 [12.3–31.4]	<0.001
Hgb>11g/dl		9.99 [4.98–15.00]	<0.001
Adhered		5.20 [0.68–9.73]	0.024
**Vitality**			
CKD stages	1 & 2	1.00	
	3	-1.8 [-7.4–3.8]	0.528
	4	-12.7 [-19.5-(-5.9)]	<0.001
	5	-14.4 [-20.0-(-8.8)]	<0.001
Family income status	Very low	1.00	
	Low	16.7 [10.1–23.4]	<0.001
	Average	23.8 [18.0–28.9]	<0.001
	Above average	26.7 [20.1–33.3]	<0.001
	High	28.3 [19.2–37.4]	<0.001
Hgb>11g/dl		7.90 [3.26–12.55]	0.001
**Social functioning**			
Occupation	Farmer	1.00	
	Gov.t employee	0.5 [-8.3–9.3]	0.909
	Merchants	-1.0 [-13.5–11.5]	0.875
	Daily laborer	-6.9 [-17.0–3.2]	0.178
	House wife	-2.9 [-13.4–7.6]	0.585
	Retired	-0.6 [-12.7–11.2]	0.922
	Others*	2.4 [1.2–4.4]	0.03
CKD stages	1 & 2	1.00	
	3	-0.6 [-8.6–7.4]	0.879
	4	-13.9 [-23.0-(-4.8)]	0.003
	5	-23.2 [-31.1-(-15.2)]	<0.001
Family income status	Very low	1.00	
	Low	15.4 [5.4–25.3]	0.003
	Average	27.5 [18.6–36.4]	<0.001
	Above average	27.2 [17.7–36.7]	<0.001
	High	33.3 [20.2–46.4]	<0.001
Hgb>11g/dl		9.15 [2.25–16.06]	0.01
**Role emotional**			
Family income status	Very low	1.00	
	Low	-0.4 [-17.5–16.7]	0.963
	Average	28.2 [10.7–45.6]	0.002
	Above average	40.6 [24.5–56.6]	<0.001
	High	30.2 [5.2–55.3]	0.019
Hgb>11g/dl		36.11 [23.74–48.48]	<0.001
**Mental health**			
Absence of complications		6.19 [2.12–10.26]	0.003
Family income status	Very low	1.00	
	Low	12.7 [5.5–5.19.8]	0.001
	Average	19.6 [13.7–25.5]	<0.001
	Above average	24.3 [18.3–30.2]	<0.001
	High	21.4 [13.2–29.6]	<0.001
Hgb>11g/dl		7.74 [3.02–12.45]	0.001

CI = confidence interval, CKD = chronic kidney disease, Hgb = hemoglobin

*students, garage, guargds, teacher working in private schools

The present study revealed that only high family income, educational status and hemoglobin >11g/dl level were predictors of better QoL in the physical component summary, after adjustments through multivariable linear regression. The independent predictors of higher mental component summary were high family income, hemoglobin >11g/dl level and absence of CKD complications (Table 9).

**Table 9:** Adjusted analysis for predictors of physical and mental component summaries of the Short Form (SF-36) among chronic kidney disease patients.

SF-36 component summary			Coefficients (95% CI)	p-value*
**Physical component summary**				
**Educational status**	Cannot read & write		Reference	
	Primary		4.0 [-0.01–9.06]	0.051
	Secondary		3.6 [-0.49–7.71]	0.083
	Higher education		7.9 [4.10–11.66]	<0.001
**Family income status**	Very low		Reference	
	Low		3.76 [0.71–6.81]	0.016
	Average		11.97 [8.83–15.12]	<0.001
	Above average		13.56 [10.68–16.43]	<0.001
	High		15.33 [11.33–19.33]	<0.001
**Hgb>11 g/dl**			8.36 [6.31–10.41]	<0.001
**Mental component summary**				
**Family income status**		Very low	Reference	
		Low	5.45 [1.87–9.03]	0.003
		Average	9.15 [5.74–12.56]	<0.001
		Above average	11.50 [8.22–14.79]	<0.001
		High	10.10 [5.10–15.10]	<0.001
**Absence of complications**			2.75 [0.56–4.94]	0.014
**Hgb>11 g/dl**			4.54 [2.01–7.08]	0.001

CI = confidence interval.

## Discussion

The present study revealed that QoL decreased across all CKD stages, which is similar with various studies [13, 21]. Quality of life domains such as physical functioning, bodily pain, vitality and social functioning were found statistically significant with CKD stages. The domains which make up the physical QoL were more impaired than domains that constitute the mental QoL. This finding is in agreement with the results obtained from previous studies, which demonstrated poorer physical component QoL in relation to mental component QoL in renal patients [5, 13]. This could probably be due to the chronic nature of the disease; patients may adapt not only to the disease and its treatment but also psychologically to their situations that directly affect patients’ QoL over time. The mean score of physical component summary was found to be 38.1±9.5. This finding is similar with previous findings in Iran [11], Canada & Denmark [22, 23] and different from other studies in Brazil [13]. On the other hand, mental composite summary was found to be 46±9.1. This finding is also in accordance with studies conducted in Brazil & Denmark [13, 23] and different from studies conducted in Nigeria & USA [10, 24]. This variation could be attributed to differences in socioeconomic and education status, management approaches between the countries, and sample size between studies. Socioeconomic status has been implicated in QoL in several studies of renal patients [13, 25]. CKD patients with low socioeconomic status could not afford the prescribed regimens for the management of various illness co-existed and this affects their QoL and adherence [26]. The finding of this study indicated that there were low QoL scores in all stages of CKD, which is associated with low socioeconomic status of the study participants in Ethiopia. Besides, it also demonstrated a significant decrease in QoL progressively in the different stages of renal disease based on the mean values of the SF-36 scores, which were below 70 in all dimensions. However, normal healthy populations usually have scores above this level in most studies [19].

On the evaluation of socio-demographic and clinical variables through multivariable linear regression analysis, all SF-36 domains, physical and mental component summary were found to be strongly associated with family income and hemoglobin level. In addition, an advanced stage of CKD was found a predictor of worse physical functioning, social functioning, vitality and bodily pain. These findings are in accordance with other studies in Denmark [23], Jordan [27], Saudi [3], Ireland [28], Portugal [7], and showed that socio-economic status was a predictor of QOL score. A prospective study conducted by Braga *et al* [26] revealed that higher socioeconomic level was significantly related to better QoL. Similarly, lower social status, characterized by lower education, worse financial situation, or lack of employment has also been consistently associated with impaired QoL [29]. This could be due to the fact that people with low economic status do not access effective health care due to economic constraints. This might suppress utilization of good QoL, effective health care in developing countries including Ethiopia.

Besides, various studies revealed that hemoglobin ≥11g/dl was associated with better QoL. A study in the US indicated that hemoglobin level was positively associated with QoL [24]. In different literature, the impact of hemoglobin in CKD on QoL is well described [30, 31]. This may be due to the low level of hemoglobin being associated with a greater risk of co-morbidities, which could result in fatigue and reduced physical activities, thus, decreasing QoL.

With regard to education, participants with a higher level of education had better physical component summary than patients with lower education level. This finding is similar to other previous studies which showed that participants with higher educational level have better QoL [32, 33]. This may be due to educated participants have greater access to information about their disease, better economic conditions, and better capacity to evaluate traumatic phenomena. It is also assumed that CKD patients with higher education mainly participate in activities that require more intellectual over those that require greater physical effort. Thus, low educational status may attribute the poor QoL of CKD patients. Presences of CKD complications were found to be strongly associated with low score on mental composite summary in the present study. This is comparable to a study by Silverberg *et al* [34] & Kimel *et al* [35] where history of CVD and anemia were found to be negatively associated with QoL. The study participants in this study who have different complications scored low QoL. The possible reason for this finding may be due to CKD patients with different complications were more likely on many drugs at the same time and may also be at advanced stage of CKD [36]. All these factors may constitute great burden for the patients and invariably reduce their QoL. In the present study variables like sex, co-morbidity status, CKD stage, occupation and pill burden were not statistically significant associated with QoL, which were predictors of in various studies. This variation may be due to methodological difference and management approaches between studies in different countries.

## Limitation of the study

The cross-sectional nature of the study did not allow a follow up, which would have provided a better design for identifying the worse quality of life and contributing factors. On the other hand, the quantitative nature of the data could not properly highlight the reasons for low quality of life from the patients’ perspective, which would have been better revealed by conducting in-depth interviews or focus group discussions. The study looked at only a single facility and hence caution should be exercised in extrapolating the results.

## Conclusions

Quality of life was impaired progressively across the 5 CKD stages. The domains which make up the physical quality of life were more impaired than domains that constitute the mental quality of life. Study participants with low income and hemoglobin level were considered to have worse quality of life in both physical and mental component summaries.

## Ethical approval

Approval and permission were sought from Ethical Review Board of school of pharmacy and department of internal medicine of Addis Ababa University.

## Supporting information

S1 Annex Structured questionnaire and data abstraction format. (DOCX)

## Abbreviations

CI: Confidence Interval
CKD: Chronic Kidney Disease
CVD: Cardiovascular Disease
GFR: Glomerular Filtration Rate
KDOQI: Kidney Disease Outcomes Quality Initiative
QoL: Quality of Life
SPSS: Statistical Package for Social Sciences
TASH: Tikur Anbessa Specialized Hospital
