# Supplementary material for: Quality of life and its predictors among patients with chronic kidney disease: A hospital-based cross sectional study
Source: PLoS One. 2019 Feb 27;14(2):e0212184. doi: 10.1371/journal.pone.0212184 (PMC6392259; doi:10.1371/journal.pone.0212184)
Supplement: S1 Annex — (DOCX) [file pone.0212184.s001.docx]

S1 Annex: Structured questionnaire and Data abstraction format

**Part I: Socio-demographic Characteristics**

1. **Sex**
2. Male B. Female
3. **Age _________ Years**
4. **Marital Status**
5. Single
6. Married
7. Divorced
8. Widowed
9. **Occupation**

A. Farmer

B. Gov’t Employee

C. Merchant/Trade

D. Daily Laborer

E. House wife

F. Retired

G. Others Specify___________________

1. **Profession**

A. Health professional B. Non-health professional

1. **Educational Status**

A. Cannot read and write

B. Primary (1-8)

C. Secondary (9-12)

D. Higher Education

1. **Monthly Family Income (in ETB) _________**

A. Very Low (<600)

B. Low (601-1500)

C. Average (1501-3500)

D. Above Average (3501-5000)

E. High (>5001)

**Part II: The Kidney Disease QOL (KDQOL) – Short Form 36 questionnaire**

| 1. **In general, would you say your health is:** | Score |
| --- | --- |
| Excellent | 1 |
| Very good | 2 |
| Good | 3 |
| Fair | 4 |
| Poor | 5 |
| 1. **Compared to one year ago,** |  |
| Much better now than one year ago | 1 |
| Somewhat better now than one year ago | 2 |
| About the same | 3 |
| Somewhat worse now than one year ago | 4 |
| Much worse now than one year ago | 5 |

1. The following items are about activities you might do during a typical day. Does your health now limit you in these activities? If so, how much? (Circle One Number on Each Line)

| **Activities** | **Yes,**  **Limited a**  **Lot (1)** | **Yes,**  **Limited a**  **Little (2)** | **No, Not**  **limited at**  **All (3)** |
| --- | --- | --- | --- |
| 1. **Vigorous activities**, such as running, lifting heavy objects, participating in strenuous sports | 1 | 2 | 3 |
| 1. **Moderate activities**, such as moving a table, pushing a vacuum cleaner, bowling, or playing golf | 1 | 2 | 3 |
| 1. Lifting or carrying groceries | 1 | 2 | 3 |
| 1. Climbing **several** flights of stairs | 1 | 2 | 3 |
| 1. Climbing **one** flight of stairs | 1 | 2 | 3 |
| 1. Bending, kneeling, or stooping | 1 | 2 | 3 |
| 1. Walking **more than a mile** | 1 | 2 |  |
| 1. Walking **several blocks** | 1 | 2 | 3 |
| 1. Walking **one block** | 1 | 2 | 3 |
| 1. Bathing or dressing yourself | 1 | 2 | 3 |

1. During the past 4 weeks, have you had any of the following problems with your work or other regular daily activities **as a result of your physical health**? (Circle One Number on Each Line)

| **Activities** | **Yes (1)** | **No (2)** |
| --- | --- | --- |
| 1. Cut down the amount of time you spent on work or other activities | 1 | 2 |
| 1. Accomplished less than you would like | 1 | 2 |
| 1. Were limited in the kind of work or other activities | 1 | 2 |
| 1. Had difficulty performing the work or other activities (for example, it took extra effort) | 1 | 2 |

1. During the **past 4 weeks,** have you had any of the following problems with your work or other regular daily activities **as a result of any emotional problems** (such as feeling depressed or anxious)? **(Circle One Number on Each Line)**

| Activities | Yes | No |
| --- | --- | --- |
| 1. Cut down the amount of time you spent on work or other activities | 1 | 2 |
| 1. **Accomplished less** than you would like | 1 | 2 |
| 1. Didn't do work or other activities as **carefully** as usual | 1 | 2 |

1. During the past 4 weeks, to what extent has your physical health or emotional problems interfered with your normal social activities with family, friends, neighbors, or groups?

| Not at all | 1 |
| --- | --- |
| Slightly | 2 |
| Moderately | 3 |
| Quite a bit | 4 |
| Extremely | 5 |

1. How much bodily pain have you had during the past 4 weeks?

| None | 1 |
| --- | --- |
| Very mild | 2 |
| Mild | 3 |
| Moderate | 4 |
| Severe | 5 |
| Very severe | 6 |

1. During the past 4 weeks, how much did pain interfere with your normal work (including both work outside the home and housework)?

| Not at all | 1 |
| --- | --- |
| A little bit | 2 |
| Moderately | 3 |
| Quite a bit | 4 |
| Extremely | 5 |

These questions are about how you feel and how things have been with you **during the past 4 weeks**. For each question, please give the one answer that comes closest to the way you have been feeling. **(Circle One Number on Each Line)**

9. How much of the time during the **past 4 weeks** . . .

|  | **All of**  **the Time** | **Most of**  **the**  **Time** | **A Good**  **Bit of the**  **Time** | **Some of**  **the Time** | **A Little**  **of the**  **Time** | **None of**  **the Time** |
| --- | --- | --- | --- | --- | --- | --- |
| 1. Did you feel full of pep? | 1 | 2 | 3 | 4 | 5 | 6 |
| 1. Have you been a very nervous person? | 1 | 2 | 3 | 4 | 5 | 6 |
| 1. Have you felt so down in the dumps that nothing could cheer you up? | 1 | 2 | 3 | 4 | 5 | 6 |
| 1. Have you felt calm and peaceful? | 1 | 2 | 3 | 4 | 5 | 6 |
| 1. Did you have a lot of energy? | 1 | 2 | 3 | 4 | 5 | 6 |
| 1. Have you felt downhearted and blue? | 1 | 2 | 3 | 4 | 5 | 6 |
| 1. Did you feel worn out? | 2 | 2 | 3 | 4 | 5 | 6 |
| 1. Have you been a happy person? | 1 | 2 | 3 | 4 | 5 | 6 |
| 1. Did you feel tired? | 1 | 2 | 3 | 4 | 5 | 6 |

1. During the past 4 weeks, how much of the time has your physical health or emotional problems interfered with your social activities (like visiting with friends, relatives, etc.)? (Circle One Number)

| All of the time | 1 |
| --- | --- |
| Most of the time | 2 |
| Some of the time | 3 |
| A little of the time | 4 |
| None of the time | 5 |

1. How TRUE or FALSE is each of the following statements for you. (Circle One Number on Each Line)

|  | **Definitely**  **True** | **Mostly**  **True** | **Don't**  **Know** | **Mostly**  **False** | **Definitely**  **False** |
| --- | --- | --- | --- | --- | --- |
| 1. I seem to get sick a little easier than other people | 1 | 2 | 3 | 4 | 5 |
| 1. I am as healthy as anybody I know | 1 | 2 | 3 | 4 | 5 |
| 1. I expect my health to get worse | 1 | 2 | 3 | 4 | 5 |
| 1. My health is excellent | 1 | 2 | 3 | 4 | 5 |

## Part III: Data abstraction format

**Part a: Clinical data (Scr, BUN, FBS, BP, CKD stage, etc.)**

1. Fasting blood sugar value (mg/dl) _____________
2. Serum creatinine _______________
3. Blood urea nitrogen _______________
4. Serum albumin _________________
5. Hemoglobin ______________
6. Blood pressure__________________
7. GFR_________________
8. CKD stage ___________________

**Part b፡ Co-morbidities and CKD Complications**

1. Presence of co morbidities

A. Present B. Absent

1. If the response for the above question is present, which of the following co-morbidity is present? (Can tick more than once)
   1. Diabetes mellitus
   2. Hypertension
   3. Ischemic Heart Disease
   4. Dyslipidemia
   5. Peripheral Vascular disease
   6. Obesity
   7. Anemia
   8. Others, Specify__________
2. Presence of CKD complications

A. Present B. Absent

1. If the response for the above question is present, which of the following CKD complication is present? (Can tick more than once)
2. Heart disease
3. Osteodystrophy
4. Anemia
5. Hyperkalaemia
6. Fluid buildup
7. Others, specify ______________

**Amharic (local language versions)**

# ምዕራፍ አንድ፡ ቃለ መጠይቅ

**ክፍል 1 ፡ አጠቃላይ መገለጫዎች**

1. ጾታ ሀ. ወንድ ለ. ሴት
2. እድሜ _________ ዓመት(በቁጥር ይፃፍ)
3. የጋብቻ ሁኔታ

ሀ. ያላገባ/ች/

ለ. ያገባ/ች/

ሐ. የተፋታ/ች/

መ. ባል የሞተባት(ሚስት የሞተችበት

4. የስራ ሁኔታ

ሀ. አርሶ አደር

ለ. የመንግስት ሰራተኛ

ሐ. ነጋዴ

መ. የቀን ሰረተኛ

ሠ. የቤት እመቤት

ረ. ጡረተኛ

ሰ. ሌላ ከሆነ ይገለጽ___________

**5.** የሙያ ሁኔታ

ሀ. የጤና ባለሙያ ለ. የጤና ባለሙያ ያልሆነ

**6**. የትምህርት ደረጃ

ሀ. ያልተማረ/ች/

ለ. አንደኛ ደረጃ (1-8)

ሐ. ሁለተኛ ደረጃ (9-12)

መ. ከፍተኛ ደረጃ(ኮሌጅ፣ ዩኒቨርሲቲ)

7. ወርሀዊ የቤተሰብ ገብ (በብር) _________

ሀ. በጣም ዝቅተኛ (<600)

ለ. ዝቅተኛ (601-1500)

ሐ. መካከለኛ (1501-3500)

መ. ከአማካይ በላይ (3501-5000)

ሠ. ከፍተኛ (>5001)

**ምዕራፍ 2 ቃለ መጠየቅ: የኩላሊት ህመምተኞች የአኗኗር ሁኔታ**

| 1. በአጠቃላይ የጤናዎ ሁኔታ ምን ይመስላል | ነጥብ |
| --- | --- |
| እጅግ በጣም ጥሩ | 1 |
| በጣም ጥሩ | 2 |
| ጥሩ | 3 |
| መካከለኛ(በቂ) | 4 |
| ጥሩ አይደለም | 5 |
| 1. ጤናዎ ከአንድ ዓመት በፊት ከነበረበት ጋር ሲነጻጸር ምን ይመስላል |  |
| በጣም የተሻለ ነው | 1 |
| የተወሰነ የተሻለ ነው | 2 |
| ተመሳሳይ ነው | 3 |
| የተወሰነ የተባባሰ የጤና ችግር አለ | 4 |
| በጣም የተባባሰ የጤና ችግር አለ | 5 |

1. የሚከተሉት በሆነ ቀን ሊሰሯቸው የሚችሉ እንቅስቃሴዎች (ስራዎች) ናቸው፡፡ እነዚህን ስታከናውኑ ያጋጠመዎት የጤና ውሱንነት ነበር? አጋጥሞዎት ከነበር ምን ያህል? (በእያንዳንዱ መስመር አንዱን ቁጥር ያክብቡ)

| እንቅስቃሴዎች | አዎ፣ ብዙ ውስንነት አጋጥሞኛል (1) | አዎ፣ የተወሰነ ውስንነት አጋጥሞኛል (2) | የለም፣ ምንም ዓይነት ውስንነት አላጋጠመኝም (3) |
| --- | --- | --- | --- |
| ሀ) አድካሚ እንቅስቃሴዎች ለምሳሌ ሩጫ፣ ከባድ እቃዎችን ማንሳት፣ በአስቸጋሪ ስፖርታዊ እንቅስቃሴዎች መሳተፍ | 1 | 2 | 3 |
| ለ) መካከለኛ እንቅስቃሴዎች ለምሳሌ፣ ጠረንጴዛ ማንቀሳቀስ፣ | 1 | 2 | 3 |
| ሐ) የምግብ ሸቀጣሸቀጦችን ሲሸከሙ | 1 | 2 | 3 |
| መ) ብዙ ደረጃዎች ሲወጡ | 1 | 2 | 3 |
| ሠ) አንድ ደረጃ ብቻ ሲወጡ | 1 | 2 | 3 |
| ረ) ሲያዘነብሉ፣ ሲንበረከኩ ወይም ሲያጎነብሱ | 1 | 2 | 3 |
| ሰ) ከአንድ ማይል (1.6ኪሎ ሜትር) በላይ ሲራመዱ | 1 | 2 | 3 |
| ሸ) ብዙ መሰናክሎችን ሲራመዱ | 1 | 2 | 3 |
| ቀ) አንድ መሰናክል ብቻ ሲራመዱ | 1 | 2 | 3 |
| በ) ገላዎትን ሲታጠቡ ወይም ልብስ ሲለብሱ | 1 | 2 | 3 |

4. ባለፉት 4 ሳምንታት ውስጥ ከአካላዊ ጤንነት ችግሮች የተነሳ ስራዎትን ወይም የእለት ተእለት እንቅስቃሴዎትን በሚያከናውኑበት ወቅት የሚከተሉት ውስንነቶች አጋጥሞዎት ነበር? (በእያንዳንዱ መስመር አንድ ቁጥር ብቻ አክብቡ)

| እንቅስቃሴዎች | አዎ(1) | የለም (2) |
| --- | --- | --- |
| ሀ. ለስራ የሚያውሉት የጊዜ መጠን በፊት ከሚጠቀሙት ያነሰ ነው? | 1 | 2 |
| ለ. የሚፈጽሙት ማከናወን ከሚፈልጉት በታች ነው? | 1 | 2 |
| ሐ. ስራ ለመስራት ወይም ለሌሎች እንቅስቃሴዎች ውሱንነት አጋጥሞዎታል? | 1 | 2 |
| መ. ስራ መስራት ወይም ሌሎች ተግባራትን ማከናወን ያለመቻል ችግር አጋጥሞዎታል(ለምሳሌ ሌላ ተጨማሪ ጥረትና እገዛ አስፈልጎዎታል) | 1 | 2 |

5. ባለፉት 4 ሳምንታት ውስጥ ከአእምሯዊ ጤንነት ችግሮች የተነሳ ስራዎትን ወይም የእለት ተእለት እንቅስቃሴዎትን በሚያከናውኑበት ወቅት የሚከተሉት ውስንነቶች አጋጥሞዎት ነበር? ለምሳሌ የድብርት ስሜት፣ መሸበር (በእያንዳንዱ መስመር አንድ ቁጥር ብቻ አክብቡ)

| **እንቅስቃሴዎች** | **አዎ(1)** | **የለም (2)** |
| --- | --- | --- |
| ሀ. ለስራ የሚያውሉት የጊዜ መጠን በፊት ከሚጠቀሙት ያነሰ ነው ? | 1 | 2 |
| ለ. የሚፈጽሙት ማከናወን ከሚፈልጉት በታች ነው? | 1 | 2 |
| ሐ. ስራዎትን ወይም ሌሎች እንቅስቃሴዎችን እንደተለመደው በጥንቃቄ ለመስራት ውሱንነት አጋጥሞዎታል? | 1 | 2 |

6. ባለፉት 4 ሳምንታት ውስጥ የአካላዊ ጤንነትና አእምሯዊ ችግሮች ከቤተሰብ፣ ጓደኛ፣ ጎረቤት፣ ወዘተ ጋር በሚያደርጉት ማህበራዊ እንቅስቃሴ ዙሪያ ተጽእኖ(እንቅፋት) ነበሩ?

| በፍፁም | 1 |
| --- | --- |
| በጣም በትንሹ | 2 |
| በትንሹ | 3 |
| በመጠኑ | 4 |
| እጅግ በጣም | 5 |

1. ባለፉት 4 ሳምንታት ውስጥ ምን ያህል አካላዊ ህመም አጋጥሞዎት ነበር?

| ምንም አላጋጠመኝም | 1 |
| --- | --- |
| በጣም ቀላል | 2 |
| ቀላል | 3 |
| መካከለኛ | 4 |
| ከባድ | 5 |
| በጣም ከባድ | 6 |

1. ባለፉት 4 ሳምንታት ውስጥ ህመም ምን ያህል በመደበኛ ስራዎት ላይ እንቅፋት ሆኖበዎት ነበር (የቤት ውስጥና ከቤት ውጭ ያሉ ስራዎችን ጨምሮ)?

| ምንም | 1 |
| --- | --- |
| በጣም በትንሹ | 2 |
| በትንሹ | 3 |
| በመጠኑ | 4 |
| በከፍተኛ | 5 |

ከዚህ በታች ያሉት ጥያቄዎች ባለፉት 4 ሳምንታት ውስጥ ነገሮችን እንዴት አእንዳሳለፏቸውና የተሰማዎት ስሜት ምን እንደነበረ ለማወቅ የተዘጋጁ ናቸው፡፡ (በእያንዳንዱ መስመር አንድ ቁጥር ብቻ አክብቡ)

| ለምን ያህል ጊዜ | ሁልጊዜ | ብዙ ጊዜ | አብዘሀኛውን ጊዜ | ለተወሰነ ጊዜ | ለትንሽ ጊዜ | ምንም |
| --- | --- | --- | --- | --- | --- | --- |
| ሀ. ሙሉ ደስተኛ ሆነው ነበር? | 1 | 2 | 3 | 4 | 5 | 6 |
| ለ. ተጨንቀው ነበር? | 1 | 2 | 3 | 4 | 5 | 6 |
| ሐ. ምንም የሚያስደስተኝ ነገር የለም ብለው አስበው ነበር? | 1 | 2 | 3 | 4 | 5 | 6 |
| መ. ሰላም ያለውና ፀጥ ያለ ስሜት ተሰምቶዎት ነበር? | 1 | 2 | 3 | 4 | 5 | 6 |
| ሠ. የጥንካሬ ስሜት ነበረዎት? | 1 | 2 | 3 | 4 | 5 | 6 |
| ረ. የትካዜ ስሜት ነበረዎት? | 1 | 2 | 3 | 4 | 5 | 6 |
| ሰ. አልፎብኛል፣ ተስፋ የለኝም፣ አልቆልኛል የሚል ስሜት ነበረበዎት? | 2 | 2 | 3 | 4 | 5 | 6 |
| ሸ. ደስተኛ ሰው ነበርክ/ሽ/? | 1 | 2 | 3 | 4 | 5 | 6 |
| ቀ. የድካም ስሜት ነበረብህ/ሽ/? | 1 | 2 | 3 | 4 | 5 | 6 |

9. ባለፉት 4 ሳምንታት ጊዜ ውስጥ ምን ያህል ፡-

1. ባለፉት 4 ሳምንታት ውስጥ የአካላዊ ጤንነትና አእምሯዊ ችግሮች ከቤተሰብ፣ ጓደኛ፣ ጎረቤት፣ ወዘተ ጋር በሚያደርጉት ማህበራዊ እንቅስቃሴ ዙሪያ ለምን ያህል ጊዜ ተጽእኖ(እንቅፋት) ነበሩ(ለምሳሌ ጓደኞችዎንና ቤተሰበዎን ለመጠየቅ)?(አንዱን ብቻ ይምረጡ)

| ሙሉውን ጊዜ | 1 |
| --- | --- |
| ብዙውን ጊዜ | 2 |
| አንዳንድ ጊዜ | 3 |
| አልፎ አልፎ | 4 |
| ምንም | 5 |

1. ለእርሰዎ የሚከተሉት ሀሳቦች ምን ያህል እውነት እና ሀሰት ናቸው (በእያንዳንዱ መስመር አንድ ቁጥር ብቻ አክብቡ)

|  | በትክክል እውነት ነው | በአብዘሀኛው እውነት ነው | አላውቀውም | በአብዘሀኛው ስህተት ነው | በትክክል ሀሰት ነው |
| --- | --- | --- | --- | --- | --- |
| ሀ. እኔ እንደሌሎቹ ሰዎች ህመም አይጠናብኝም | 1 | 2 | 3 | 4 | 5 |
| ለ. እኔ እንደማውቃቸው ሰዎች ሁሉ ጤነኛ ነኝ | 1 | 2 | 3 | 4 | 5 |
| ሐ. የኔ ጤና ችግር ይገጥመዋል ብየ እጠብቃለሁ | 1 | 2 | 3 | 4 | 5 |
| መ. የእኔ ጤና እጅግ በጣም ጥሩ ነው | 1 | 2 | 3 | 4 | 5 |
